# Models suggest pathogen risks to wild fish can be mitigated by acquired immunity in freshwater aquaculture systems

**DOI:** 10.1038/s41598-020-64023-2

**Published:** 2020-05-05

**Authors:** Mickael Teixeira Alves, Nick G. H. Taylor

**Affiliations:** Centre for Environment, Fisheries and Aquaculture Science (Cefas), Barrack Road, Weymouth Dorset, DT4 8UB UK

**Keywords:** Ecological modelling, Population dynamics

## Abstract

The interaction of pathogens between wild and farmed aquatic animal populations is a concern that remains unclear and controversial. *Ichthyophthirius multifiliis*, a ciliated protozoan parasite, is a pathogen of freshwater finfish species with geographic and host range that causes significant economic losses in aquaculture. Flow-through farming systems may facilitate the transfer of such a parasite with free-living stages between farmed and wild stocks. Here, experimental and field study infection data are used to describe the infection dynamics of *Ichthyophthirius multifiliis* in rainbow trout using a simple macroparasite model by including host resistance. The study considered flow-through farming systems with a single or two age-class compartments and simulated the transfer of the parasite between farmed and wild fish populations. Results suggest that aquaculture can promote the prevalence of the resistance in wild stocks by increasing the parasite population in the wild environment. At the same time, acquired resistance in the farmed fish population may protect the wild fish population from lethal effects of the parasite by reducing the total parasite population. This study offers a promising mathematical basis for understanding the effects of freshwater aquaculture in disease spread in wildlife, developing risk assessment modeling, and exploring new ways of aquaculture management.

## Introduction

Aquaculture production has rapidly increased for the last four decades and is considered a key solution to meet the world food demand^1^. At the same time, concerns have been raised about environmental impacts, including infectious diseases in wild fish^2–5^. However, most examples of pathogen interactions between farmed and wild fish derive from marine systems, focusing largely on: the salmon louse (*Lepeophtheirus salmonis*), a parasite that infects wild and farmed salmonids^6–8^, white spot syndrome virus (WSSV), a pathogen found in wild and farmed shrimp^9–11^, and *Anguillicoloides crassus*, a nematode that emerged in wild European eels^12^.

Though declines in wild marine stocks have been associated with aquaculture sites introducing or amplifying pathogens^13–17^, such interactions have received little study in freshwater ecosystems^3,18–20^. Yet, pathogen transfer from farmed to wild stocks, termed ‘spillover’, may be facilitated by commonly used flow-through farming systems in which water is often abstracted from rivers before being discharged back to the river after passing through the farm site^21^. The potential impact of freshwater aquaculture on wild fish populations however remains unclear and controversial^22,23^.

Through a mathematical modeling approach, this study aimed to investigate the potential interactions between aquaculture systems and wild fish stocks based on an important pathogen of freshwater finfish aquaculture, *Ichthyophthirius multifiliis*, otherwise known as ‘Ich’. This is a ciliated protozoan parasite that is globally distributed and causes disease in a wide range of freshwater finfish species, but is of significant concern in trout, carp and catfish aquaculture^24^. Ich causes significant economic losses to these sectors through direct mortality and treatment costs^25^. The parasites life-cycle includes free-living stages that can easily be transferred between farms and wild environments.

Aquaculture systems are known to amplify the numbers of this parasite substantially^26^, thus potentially increasing the exposure risk to wild fish. However, fish surviving infection by this parasite become resistant to subsequent infections by acquiring a long-term protective immunity via innate and adaptive immune responses^27,28^ and hence constitute dead-end contacts for the parasite^29^. It may therefore be possible for farm sites to mitigate risks to wild stocks by using fish surviving exposure to the parasite to interrupt the parasite’s life cycle and remove it prior to exiting the farm site.

The mechanisms involved in disease transfer have been theoretically explored within marine ecosystems with classical microparasite models^30,31^, macroparasite models^32^, spatial models^33,34^ and hydrodynamic models^35,36^. The dynamics of fish populations exposed to Ich have also been described using simple deterministic models^24,37–39^. The present study aimed to explore the effects of Ich transferred between wild freshwater finfish and farmed rainbow trout populations through an extended macroparasite model^40^ by including host resistance. The study focuses on the effects of Ich on mortality, resistance (via acquired immunity) and persistence of wild and farmed fish, specifically exploring how host immunity to Ich and different farming systems influence fish dynamics and parasite outbreaks. In a more general context, this article provides a general adaptable theoretical framework that could be applied to a wide range of aquaculture systems to aid in disease management and impact assessment.

## Results

**Figure 1:**
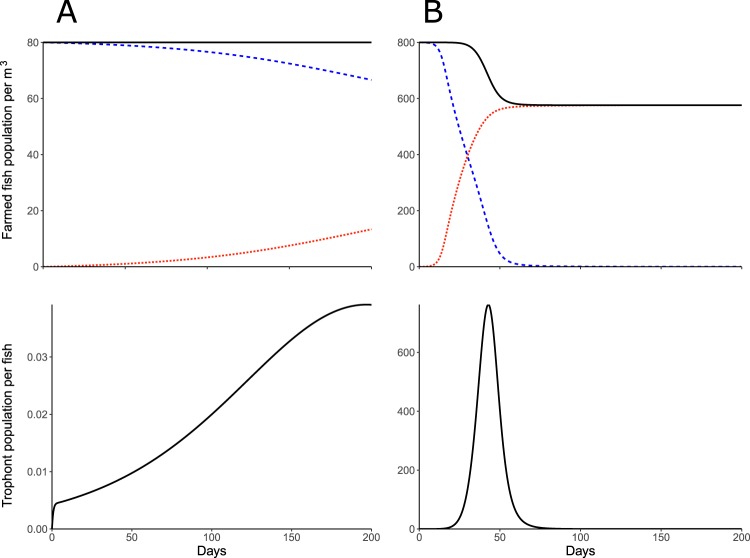
Farmed fish population (top) and trophont population (bottom) in time (day) for the deterministic continuous model (1) with initial farmed fish populations per cubic meter fixed at (**A**) ${S}_{{0}_{f}}=80$ and (**B**) ${S}_{{0}_{f}}=800$. The black lines are the total farmed fish population and the total trophont population per fish, the blue dashed lines are the susceptible farmed fish population and the orange dotted lines are the resistant farmed fish population. Parameters are in Table 1.

Model results suggest that in the absence of the parasite, the farmed fish population remains constant over the production period, assuming no natural mortality during that period. In the presence of the parasite, the farmed-fish model shows that the mortality due to the infection is dependent on the density of rainbow trout. Based on experimental parameter estimates and simulations, and assuming a low density of 80 farmed fish per cubic meter, representative of naturally-occurring densities in wild stocks, or a more realistic industrial density of 800 farmed fish per cubic meter^26,41^, the parasite either increases and induces immunity in the fish population (16.68% immunity) with no effect on the fish survival, or increases mortality in the fish population (27.38% total mortality) respectively (Fig. 1). In the latter scenario, the majority of the surviving population becomes resistant (99.98%), which subsequently prevents further persistence of the parasite.

**Figure 2:**
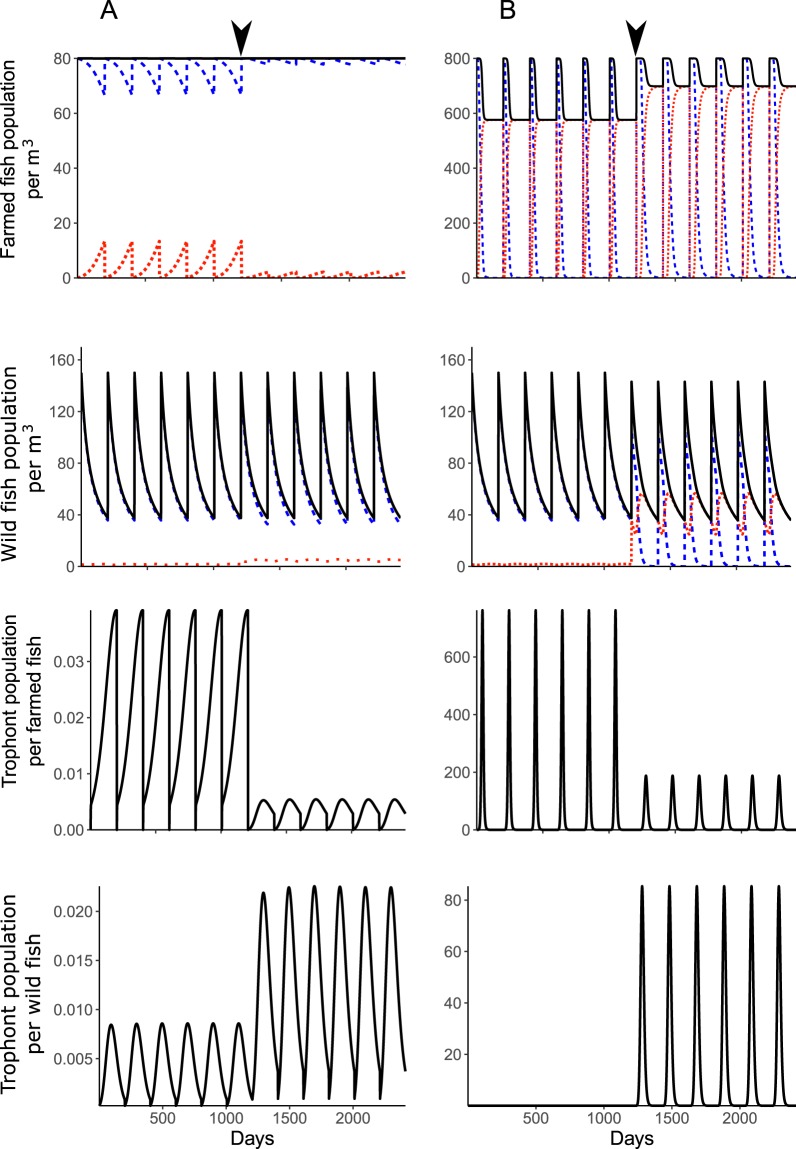
From top to bottom, farmed fish population per cubic meter, wild fish population per cubic meter, trophont population per farmed fish and trophont population per wild fish in time (day) for the deterministic semi-discrete model (3) with (**A**) 80 initial farmed fish per cubic meter and (**B**) 800 initial farmed fish per cubic meter. Ich transfer between farmed fish and wild fish starts at *t* = 1200 days and is represented by a black arrow. The black lines are the total fish or trophont populations, respectively, the blue dashed lines are the susceptible fish populations and the orange dotted lines are the resistant fish populations.

When not interacting with the farmed fish population, and in the absence of the parasite, the wild fish population reaches stable limit cycles due to the discrete reproduction term (not shown). In the presence of the parasite, the wild fish population continues to oscillate (Fig. 2). Increased parasite numbers occur in the wild fish at each host reproductive cycle, this does not cause any additional mortality, but induces resistance in a small proportion of the wild fish population (3.45% at the end of a reproductive cycle). Susceptible fish provide a natural reservoir for the parasite, maintaining it in the wild fish population and allowing them to initiate farm infections as naïve stocks are introduced.

**Figure 3:**
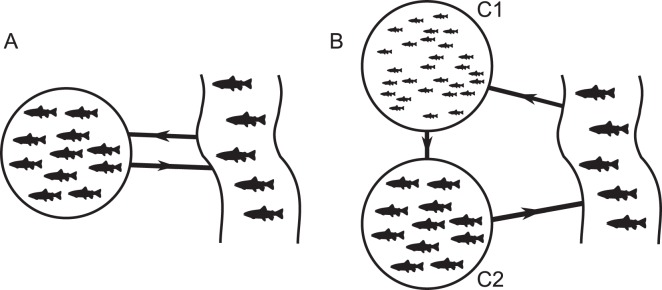
Flow-through farming system (left) with water exchange with external water sources (right). (**A**) a flow-through system with a single age-class compartment, (**B**) a flow-through system with 2 age-class compartments (C1: juveniles and C2: adults).

The interaction between farmed and wild fish populations is simulated with a continuous two-way transfer of the parasite between both environments (Fig. 3A). At a low density of farmed fish, the continuous transfer of the parasite between the farm unit and the environment does not substantially affect the wild fish population density, but increases the proportion of resistant wild fish at the end of the reproduction cycle (13.17%) (Fig. 2A). In contrast, the parasite pressure on farmed fish is reduced due to a lower infection rate, which also reduces the prevalence of resistance in the farmed population (2.71%). A higher density of farmed fish is associated with an increased rate of the parasite population being transferred to the wild fish population (Fig. 2B). This induces a substantial increase in the resistant proportion of the wild fish population (99.79%), but also increases mortality (4.67%). In contrast, the infection pressure is reduced in the farm due to the large proportion of parasites transferred to the wild fish, causing mortality to substantially decrease (12.66%) whilst maintaining a very high prevalence of resistance at the end of the production cycle (99.85%).

**Figure 4:**
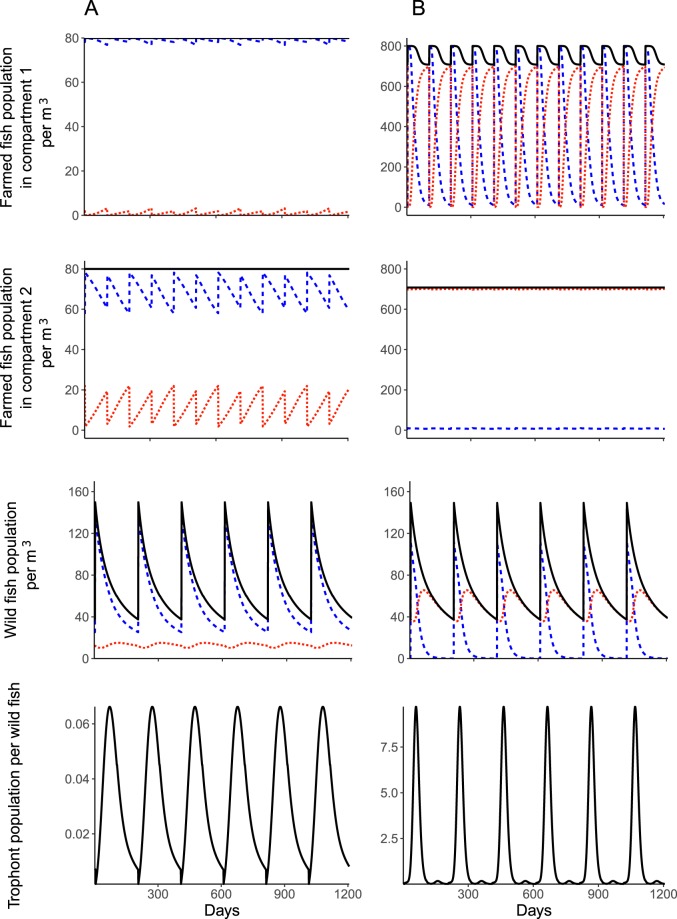
From top to bottom, farmed fish population per cubic meter in compartment 1, farmed fish population per cubic meter in compartment 2, wild fish population per cubic meter, and trophont population per wild fish in time (day) for the deterministic semi-discrete model (3) adapted to a farming system with two age-class compartments and with (**A**) 80 initial farmed fish per cubic meter in compartment 1, and (**B**) 800 initial farmed fish per cubic meter in compartment 1. The black lines are the total fish or parasite population, respectively. The blue dashed lines are the susceptible fish population and the orange dotted lines are the resistant fish population. Parameters are in Table 1.

Typical freshwater trout aquaculture systems tend to hold multiple age-classes of fish (as opposed to single age class that are all stocked and then harvested at the same time), often with older age classes relying on second use water that has already passed through compartments containing younger fish (Fig. 3B). In this case, at a low density of farmed fish, the parasite does not induce any mortality either in the wild fish population or the farmed fish populations from each compartment (Fig. 4A). The prevalence of resistance does however substantially increase in the wild fish population (32.55% at the end of the reproduction cycle) and in the farmed fish population (2.28% in compartment 1 and 27.42% in compartment 2 at the end of the production cycle). At the higher density of farmed fish, the regular introduction of susceptible juvenile farmed fish increases the frequency of outbreaks of the parasite in the farm (Fig. 4B). As a result, this is found to promote more epidemic peaks in wild fish, characterized by a succession of high and low epidemic peaks that are linked to the introduction of naïve stocks into the farm at the beginning of, and during the reproduction cycle of the wild fish respectively. The proportion of resistant wild fish consequently increases (99.93%), but no additional mortality is predicted. Acquired immunity reduces the parasite density in the farm, causing mortality in farmed fish to decrease and vary slightly in alignment with the level of the epidemic peak in the wild fish population (farmed fish mortality of 11.54% and 11.51%, for high or small epidemic peaks in the wild fish population, respectively). The prevalence of resistance in the farmed fish population remains very high at the end of the production cycle (99.09%).

Elasticity analysis confirmed the robustness of the results with the ratio of proportional change between parameter value and farmed and wild fish populations being lower than 1 for the majority of parameters. The mean time for a fish to acquire immunity *s*_*g*_ had the strongest influence on the farmed fish dynamics: the elasticity was 0.76 in the farmed fish population in model (1) and 0.56 and 0.57 in model (3) with a single or two age-classes, respectively. The wild fish population was not sensitive to any change of parameters (elasticity lower than 0.25 for most parameters) except the competition rate *α* that directly conditions the density of the total population and induced an elasticity of 1.01.

## Discussion

Worldwide, aquaculture production is expected to increase by 61.9% over the period of 2010 to 2030^1^. Such an increase raises concerns regarding disease related impacts on wildlife populations^3,34^. Due to the transfer of pathogens between wild and farmed fish populations, aquaculture could result in serious ecological and economical problems on both wild and farmed fish if not assessed and managed carefully^5^. Existing research has however reached contradictory conclusions about the impact of aquaculture on the occurrence of disease in wild fish^3^. The present study explores the potential interactions between a key pathogen of farmed and wild fish in freshwater systems using a simple deterministic semi-discrete modeling framework that assumes fish can become resistant to infection due to acquired immunity^29,39^. The study highlights i) the contribution of farmed fish in promoting resistance to Ich in wild fish populations, ii) the influence of farm husbandry practices on the parasite dynamics, and iii) the absence of mortality in the wild fish population due to parasite transfer from modern freshwater fish farms.

The two-way transfer of the parasite between farmed and wild stocks can affect immunity in the wild fish population. Here, an outbreak in the farm always induces an increase of the parasite in the wild population due to spillover, but as this exposure occurs at low levels, immunity in the wild fish population increases rather than mortality. There is a large body of evidence demonstrating dependence between acquired immunity in fish and environmental factors^42–46^. This study suggests that farmed fish may also strongly influence the immune response of wild fish. In the model, farmed fish and wild fish interact through the transfer of the parasite, which induces changes in the density of the parasite contacting the wild fish population. This is reflected by the close association between the increase in the prevalence of immunity in the wild fish population and the density of the farmed fish population which determines the parasite abundance on the farm, and, thus, the number of parasites transferred to the wild fish population. The increase in the parasite density eventually increases the exposure to the parasite which, in turn, affects the immune response^29,47^.

Farming systems influence the transfer of the parasite and alter host-parasite dynamics in modifying the density of the parasite. Effects of farming systems on water quality, fish production and environmental pollution have been largely been explored in freshwater and marine environments^48–51^, but little attention has been paid to identifying the influence of farming systems on fish disease^52,53^. This was addressed in the present study by modeling flow-through farms with a single age-class compartment or multiple age-class compartments. The single age-class system always increased the abundance of the parasite in the wild fish population, irrespective of the density of farmed fish, and could cause a small increase in mortality of the wild fish population. The multi age-class system causes the prevalence of resistance in farmed fish to increase with age-class. This had the effect of reducing the transfer of the parasite to the wild fish population and did not cause any additional mortality in wild stocks, though the frequency of epidemic peaks increased in the wild fish population as a direct consequence of the repetitive stocking of susceptible farmed fish which caused pulses in the numbers of parasites discharged into the wild environment. In contrast, the wild fish population always acts as a reservoir of the parasite^13^ and thus contributes to continuously infect naive farmed stocks through spillback facilitated by both farming systems.

Aquaculture has long been suspected to cause decline and extinction of wild fish in marine environments^17,54–56^. This study however indicates that in this case study the transfer of the parasite between farmed and wild fish is unlikely to cause such effects on freshwater wild fish populations. The models applied in this study assume a worst case scenario where conditions are likely to promote outbreaks of the parasite, including optimal temperature for the parasite with no seasonality^25^, high-densities of farmed and wild fish populations^57^ and continuous flow-through system which will facilitate continuous spillover. The fish farm with a single age-class compartment had little effect on the mortality of the wild fish population that continued to oscillate with a smaller amplitude. The more realistic scenario involving a farming system with multiple age-class compartment indicated that the transfer of the parasite between farms and wild fish populations did not induce any increase in mortality of the latter. In this study system, stocking of new susceptible farmed fish in the first age-class amplified the parasite increasing exposure to the second compartment in which most of the fish are resistant and act as a filter, removing the parasite before exposure to the environment occurs. To our knowledge, this is the first time it has been shown that immunity in farmed fish may mitigate the effect of parasites on freshwater wild fish.

For the purpose of this study it is assumed that fish mix homogeneously within their respective populations. Although this is expected to be true for farmed fish which are produced at relative high densities and are continually mixing, wild populations are likely to be more aggregated in their distribution and interact in more complex ways^58^. As wild fish inhabit a larger area, parasite infection dynamics and their impacts will vary much more than observed in farm populations due to environmental conditions and exposure to stressors such as predation^59^. Here the wild fish population was assumed to be strongly aggregated and highly-dense around farms^60,61^. Future extensions to this modeling study may wish to consider the potential consequences of spatially aggregated and connected wild fish populations in freshwater^62^ it is however likely that given the indirect route by which this particular parasite is transmitted that this will be of little consequence.

The host-parasite model assumes that environmental conditions are kept constant during the production cycle of the farmed fish. Although seasonality is known to have a significant impact on this and other endemic parasites^25^, this study focuses on a constant temperature that optimizes the development of the parasite. Extrapolation of demographic parameters from industry and laboratory studies also reflects extreme conditions in terms of infection risks of the fish populations^57^. Though these assumptions simplify the model, the approach clearly assesses the underlying mechanisms impacting the interactions between farmed and wild fish and constitutes a promising first step in the understanding of freshwater aquaculture interactions with wild ecosystems. Overall, this simple adaptation of a classical macroparasite approach^40^ paves the way to innovative and challenging models exploring spillover of other parasites in freshwater aquaculture systems.

Given the controversy about pathogen spread from aquaculture sites to wild stocks, a better understanding of the host-pathogen dynamics within and between these systems is critical, and is key to controlling disease in farmed aquatic animals and limiting any adverse impact to wild populations^3^. Simple host-parasite models have been intensively used for assessing the impact of the host density on disease outbreaks in farmed and wild fish populations, and for predicting the fate of parasite-host systems in marine environments^57,63,64^. In freshwater, factors influencing outbreaks are also numerous but remain understudied notwithstanding the importance of freshwater aquaculture and wildlife^65,66^. Despite its simplicity, the model applied in this study shows great robustness to parameter uncertainty and efficiently illuminates how the infective and free-living stages of an important freshwater parasite can alter interactions between farmed and wild fish through its transfer. In terms of management strategies, the model could be extended to represent husbandry interventions targeting specific life-stages of the parasite^26^, inform risk assessment methods^67^ and provide decision support to aquaculture safety^57^.

There is good evidence that acquired immunity has important implications in host-parasite dynamics corresponding to infection of finfish by Ich^25,39,68^. The trends described in this study indicate that farmed fish that acquire immunity can act as a biological filter for Ich by increasing the number of dead-end contacts for the parasite thus affecting the overall infection dynamics, which in turn could be used to mitigate parasite risks to wild fish. Acquired resistance can be induced by a direct exposition to the parasite^69^, but also by vaccination^70^. Herd immunization has however showed limited protection against Ich despite the promising development of vaccines over the last decades^29,71,72^. While research on immune protection of farmed aquatic animals continues, it is reassuring to note that in this case study aquaculture is unlikely to induce detrimental effects on wild fish survival.

## Methods

Exploration of the host-pathogen dynamics of Ich within and between wild and farmed fish populations was based on a well-established, flexible and adaptable macroparasite model^40^. The model was first applied to understand the dynamics of juvenile farmed rainbow trout infected by Ich^26^ and was then extended to explore the dynamics of infected wild rainbow trout. Transfer of the parasite between farmed and wild populations was achieved by modeling the two-way transfer of the parasite through water flow between the two environments. Two different flow-through farming systems (where water in the farm is continuously replaced from a water source rather than being static or recirculated through a filtration systems) were modeled with either a single age-class compartment or two age-class compartments held separately, but linked by water connectivity (as often observed in trout farming). Mortality and parasite resistance in the farmed and wild fish populations predicted by models where wild and farmed populations are connected were compared to the outputs of the individual population models.

Parametrisation of the models was based on experimental observations^26^, taken or averaged from the literature^25,29,37,39,47,68,69,73,74^, field observations^75^ and internal expertise (Table 1). Simulations were conducted under the assumption of a constant temperature (20°C) corresponding to the optimal temperature for the development of the parasite^24,47^. The farmed fish population was simulated with 800 fish per cubic meter, extrapolating the fish density from experimental data^26^. Simulations were also conducted with 80 fish per cubic meter to evaluate the impact of the fish density on the transfer of the parasite. The wild fish population was assumed to be limited by resources through intraspecific competition^76^, but could reach up to 80 fish per cubic meter. Model elasticity was performed by calculating the proportional change in the fish population that resulted from a proportional change in the parameter^77–79^:

$${\xi }_{p}=\frac{p}{N}\frac{\partial N}{\partial p},$$

with *p* a parameter and *N* the total farmed or wild fish population size at the end of the production cycle or the reproduction cycle respectively.

**Table 1:** Parameters used in farmed-fish, wild-fish and transfer models (1), (2) and (3).

Parameter	Description	Units	Value/range	Source
*s*_*g*_	mean time from exposure to resistance	*d*	25/14–28	averaged^29,39,68,69,83^
*α*	parasite-induced mortality rate	*d*^−1^	0.00025	calculated from data^26^
*λ*_*s*_	trophont residence time rate	*d*^−1^	0.1	averaged^25,37^
*β*	infection rate	*m*^3^*d*^−1^	$\frac{1}{18500}$	derived by adjusting model^26^
*c*	wild fish intracompetition rate	1	10^−4^/2.5.100^−3^−8.10^−5^	averaged and adjusted by an arbitrary dilution factor 10 for wild fish^41^
*n*	reproduction rate	1	3	calculated from literature (produced eggs, survival, sex ratio)^75^
${\mu }_{{P}_{r}}$	protomont mortality rate	*d*^−1^	1	personal observation/from literature^25,74^
${\mu }_{{C}_{y}}$	encysted tomont mortality rate	*d*^−1^	$\frac{1}{3}$	personal observation/from literature^25,74^
${\mu }_{{T}_{h}}$	theront mortality rate	*d*^−1^	1/0.3–2	averaged^25^
${\lambda }_{{P}_{r}}$	protomont residence time rate	*d*^−1^	4/4–96	personal observation/from literature^25^
${\lambda }_{{C}_{y}}$	encysted tomont residence time rate	*d*^−1^	0.8	from literature^25,73^
${\lambda }_{{T}_{h}}$	theronts produced per encysted tomont	1	500/64–1000	averaged^25,26,47,73^
*e*	daily rate of the theront transfer from wild fish to farmed fish	1	0.1	based on industry data
*d*	daily rate of the theront transfer from farmed fish to wild fish	1	0.1	based on industry data
*d*_1_	daily rate of the theront transfer from compartment 1 to 2	1	0.5	based on industry data
*d*_2_	daily rate of the theront transfer from compartment 2 to wild fish	1	0.1	based on industry data

### Model assumptions

**Figure 5:**
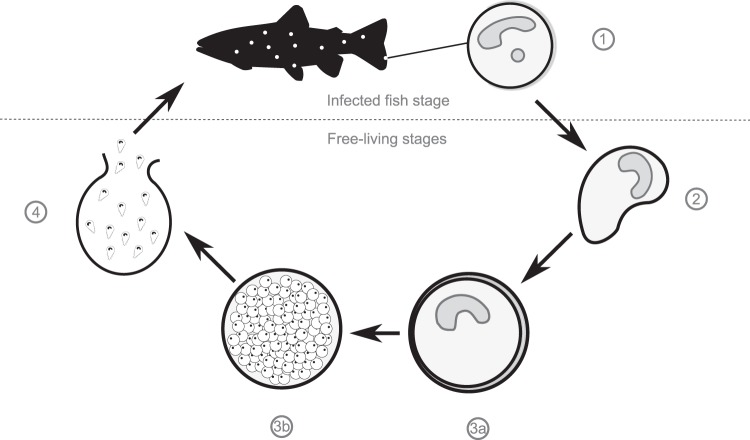
Ich life stages: (1) Trophonts feed in fish skin or gills, forming white spots (infected fish stage). (2) Protomont are released when the parasite exits fish (free-living stage). (3a) and (3b) Tomonts secrete a cyst wall and divide into infective theronts (free-living stage). (4) Theronts are released after the tomont bursts, and attach to fish to become trophonts (infective free-living stage).

The model developed in this study extends a classical macroparasite modeling approach^40^ by including host resistance and three free-living stages of parasite (Fig. 5). Fish dynamics are semi-discrete^80^, undergoing continuous dynamics most of the time and discrete harvests in fish farms and discrete reproduction in wild environments. Both types of fish population have the same interactions with the parasite, including aggregation, parasite-induced mortality, and acquired resistance to Ich. The latter may induce fitness costs that in turn impact the population dynamics^81^, however there is currently no evidence for this in this system, and investigating fitness cost was beyond the scope of this study. Freshwater flow-through farming units (in which water can be diverted from a natural environment and discharged in the same environment) were modeled by linear terms linking wild and farm populations of Ich at the theront stage, *i.e*. at the infectious free-living stage that attaches to fish (Fig. 5). The linear terms represented the spillover of Ich from farmed fish to wild fish and, in return, the reverse spillover from wild fish to farmed fish^21^ (Fig. 3).

### Farmed fish model

The model reflects industry practices by assuming that both susceptible and resistant farmed fish populations (*S*_*f*_ and *R*_*f*_, respectively) have no demographic dynamics during a production cycle of 200 days. A constant number of susceptible fish was introduced to the farm prior to each production cycle. The parasite infects fish at the theront stage (${T}_{{h}_{f}}$), and induces mortality or resistance in susceptible fish at the trophont stage (${T}_{{r}_{f}}$). Parasite stages that have exited the host corresponded to the promont (${P}_{{r}_{f}}$) and the encysted tomont (${C}_{{y}_{f}}$) stages. The model thus reads:

1 $$t\ne \tau \{\begin{array}{ccc}{\dot{S}}_{f} & = & -\frac{r}{{s}_{g}}{S}_{f}-\alpha {\lambda }_{{T}_{h}}{T}_{{r}_{f}},\\ {\dot{R}}_{f} & = & \frac{r}{{s}_{g}}{S}_{f},\\ {\dot{T}}_{{r}_{f}} & = & \beta {T}_{{h}_{f}}{S}_{f}-\alpha {\lambda }_{{T}_{h}}{T}_{{r}_{f}}\left(\frac{{T}_{{r}_{f}}}{{S}_{f}}+1\right)-\frac{r}{{s}_{g}}{T}_{{r}_{f}},\\ {\dot{P}}_{{r}_{f}} & = & {\lambda }_{{T}_{h}}{T}_{{r}_{f}}-{\mu }_{{P}_{r}}{P}_{{r}_{f}}-{\lambda }_{{P}_{r}}{P}_{{r}_{f}},\\ {\dot{C}}_{{y}_{f}} & = & {\lambda }_{{P}_{r}}{P}_{{r}_{f}}-{\mu }_{{C}_{y}}{C}_{{y}_{f}}-{\lambda }_{{C}_{y}}{C}_{{y}_{f}},\\ {\dot{T}}_{{h}_{f}} & = & {\lambda }_{{C}_{y}}{\lambda }_{{T}_{h}}{C}_{{y}_{f}}-{\mu }_{{T}_{h}}{T}_{{h}_{f}}-\beta {T}_{{h}_{f}}{S}_{f},\end{array}$$

$$t=\tau \{\begin{array}{c}{S}_{{f}_{\tau }}={S}_{{f}_{0}},{R}_{{f}_{\tau }}=0,\\ {T}_{{r}_{{f}_{\tau }}}=0,{P}_{{r}_{{f}_{\tau }}}=0,{C}_{{y}_{{f}_{\tau }}}=0,{T}_{{h}_{{f}_{\tau }}}={T}_{{h}_{{f}_{0}}},\end{array}$$

in which *r* is the probability that a fish is free of the parasite; *s*_*g*_ is the mean time from host exposure to resistance; *α* is the parasite-induced host mortality rate; ${\lambda }_{{T}_{h}}$ is the trophont exit rate from the host; *β* is the theront infection rate; ${\mu }_{{P}_{r}}$, ${\mu }_{{C}_{y}}$ and ${\mu }_{{T}_{h}}$ are the protomont, encysted tomont and theront mortality rates, respectively; ${\lambda }_{{P}_{r}}$, ${\lambda }_{{C}_{y}}$ and ${\lambda }_{{T}_{h}}$ are the rates of becoming an encysted tomont, a theront or a trophont, respectively. Though the distribution of parasites can be aggregated on fish, preliminary analysis of Ich infection in rainbow trout^26^ demonstrated that the negative binomial distribution converges to a Poisson distribution (not shown)^32,37^. Therefore, it is assumed that *r* follows a Poisson distribution that depends on the average number of trophonts per fish, and is expressed by $r=1-P({T}_{{r}_{f}}{S}_{f}^{-1})$. ${S}_{{f}_{0}}$ and ${T}_{{h}_{{f}_{0}}}$ correspond to the initial susceptible farmed fish population and number of theront introduced at the beginning of the production cycle respectively.

From model (1), the number of theronts produced by one trophont is given by:

$${\lambda }^{\ast }={\lambda }_{{T}_{h}}\left(\frac{{\lambda }_{{P}_{r}}{\lambda }_{{C}_{y}}}{({\mu }_{{P}_{r}}+{\lambda }_{{P}_{r}})({\mu }_{{C}_{y}}+{\lambda }_{{C}_{y}})}\right)\mathrm{}.$$

Given λ^*^ and a specific susceptible fish density *S*_*f*_, the basic reproduction ratio for model (1) can be written in the form

$${R}_{0}={\lambda }^{\ast }\left(\frac{\beta {S}_{f}}{\beta {S}_{f}+{\mu }_{{T}_{h}}}\right)\mathrm{}.$$

### Wild fish model

The wild fish model assumes discrete reproduction (as observed in most relevant wild fish species in temperate climates^82^) and intraspecific competition between fish^76^. Offspring was considered as susceptible. The model thus reads:

2 $$t\ne \tau \{\begin{array}{ccc}{\dot{S}}_{w} & = & -\frac{r}{{s}_{g}}{S}_{w}-\alpha {\lambda }_{{T}_{h}}{T}_{{r}_{w}}-c{S}_{w}({S}_{w}+{R}_{w}),\\ {\dot{R}}_{w} & = & \frac{r}{{s}_{g}}{S}_{w}-c{R}_{w}({S}_{w}+{R}_{w}),\\ {\dot{T}}_{{r}_{w}} & = & \beta {T}_{{h}_{w}}{S}_{w}-\alpha {\lambda }_{{T}_{h}}{T}_{{r}_{w}}\left(\frac{{T}_{{r}_{w}}}{{S}_{w}}+1\right)-\frac{r}{{s}_{g}}{T}_{{r}_{w}},\\ {\dot{P}}_{{r}_{w}} & = & {\lambda }_{{T}_{h}}{T}_{{r}_{w}}-{\mu }_{{P}_{r}}{P}_{{r}_{w}}-{\lambda }_{{P}_{r}}{P}_{{r}_{w}},\\ {\dot{C}}_{{y}_{w}} & = & {\lambda }_{{P}_{r}}{P}_{{r}_{w}}-{\mu }_{{C}_{y}}{C}_{{y}_{w}}-{\lambda }_{{C}_{y}}{C}_{{y}_{w}},\\ {\dot{T}}_{{h}_{w}} & = & {\lambda }_{{C}_{y}}{\lambda }_{{T}_{h}}{C}_{{y}_{w}}-{\mu }_{{T}_{h}}{T}_{{h}_{w}}-\beta {T}_{{h}_{w}}{S}_{w},\end{array}$$

$$t=\tau \{\begin{array}{c}{S}_{{w}_{\tau }}=n({S}_{{w}_{\tau -1}}+{R}_{{w}_{\tau -1}})+{S}_{{w}_{\tau -1}},{R}_{{w}_{\tau }}={R}_{{w}_{\tau -1}},\\ {T}_{{r}_{{w}_{\tau }}}={T}_{{r}_{{w}_{\tau -1}}},{P}_{{r}_{{w}_{\tau }}}={P}_{{r}_{{w}_{\tau -1}}},{C}_{{y}_{{w}_{\tau }}}={C}_{{y}_{{w}_{\tau -1}}},\\ {T}_{{h}_{{w}_{\tau }}}={T}_{{h}_{{w}_{\tau -1}}},\end{array}$$

in which the parameters are the same as for model (1); *c* the intraspecific competition rate; *β* the theront infection rate and *n*, the discrete reproduction component^80^. The index *f* referring to farmed fish in model (1) is replaced here with *w* referring to wild fish in the population and stage names. The parasite model does not change, so that the basic reproduction ratio for model (2) is the same as for model (1).

### Wild fish - farmed fish - parasite transfer models

In farming system A, the transfer of Ich between farmed and wild fish was assumed to be continuous and was described using additional terms in the free-living theront dynamics (model (3), Fig. 3A). It was assumed that *d*, the daily rate of theront spillover from the farmed fish to the wild fish is more important than *e*, the daily rate of the theront reverse spillover from the wild fish to the farmed fish, as water dilution is more important in wild environments. Every 200 days, farmed fish are removed and replaced with new susceptible juvenile fish.

3 $$t\ne \tau \{\begin{array}{ccc}{\dot{S}}_{f} & = & -\frac{r}{{s}_{g}}{S}_{f}-\alpha {\lambda }_{{T}_{r}}{T}_{{r}_{f}},\\ {\dot{R}}_{f} & = & \frac{r}{{s}_{g}}{S}_{f},\\ {\dot{T}}_{{r}_{f}} & = & \beta {T}_{{h}_{f}}{S}_{f}-\alpha {\lambda }_{{T}_{r}}{T}_{{r}_{f}}\left(\frac{{T}_{{r}_{f}}}{{S}_{f}}+1\right)-\frac{r}{{s}_{g}}{T}_{{r}_{f}},\\ {\dot{P}}_{{r}_{f}} & = & {\lambda }_{{T}_{r}}{T}_{{r}_{f}}-{\mu }_{{P}_{r}}{P}_{{r}_{f}}-{\lambda }_{{P}_{r}}{P}_{{r}_{f}},\\ {\dot{C}}_{{y}_{f}} & = & {\lambda }_{{P}_{r}}{P}_{{r}_{f}}-{\mu }_{{C}_{y}}{C}_{{y}_{f}}-{\lambda }_{{C}_{y}}{C}_{{y}_{f}},\\ {\dot{T}}_{{h}_{f}} & = & {\lambda }_{{C}_{y}}{\lambda }_{{T}_{h}}{C}_{{y}_{f}}-{\mu }_{{T}_{h}}{T}_{{h}_{f}}-\beta {T}_{{h}_{f}}{S}_{f}-d{T}_{{h}_{f}}+e{T}_{{h}_{w}},\\ {\dot{S}}_{w} & = & -\frac{r}{{s}_{g}}{S}_{w}-\alpha {\lambda }_{s}{T}_{{r}_{w}}-c{S}_{w}({S}_{w}+{R}_{w}),\\ {\dot{R}}_{w} & = & \frac{r}{{s}_{g}}{S}_{w}-c{R}_{w}({S}_{w}+{R}_{w}),\\ {\dot{T}}_{{r}_{w}} & = & \beta {T}_{{h}_{w}}{S}_{w}-\alpha {\lambda }_{s}{T}_{{r}_{w}}\left(\frac{{T}_{{r}_{w}}}{{S}_{w}}+1\right)-\frac{r}{{s}_{g}}{T}_{{r}_{w}},\\ {\dot{P}}_{{r}_{w}} & = & {\lambda }_{s}{T}_{{r}_{w}}-{\mu }_{{P}_{r}}{P}_{{r}_{w}}-{\lambda }_{{P}_{r}}{P}_{{r}_{w}},\\ {\dot{C}}_{{y}_{w}} & = & {\lambda }_{{P}_{r}}{P}_{{r}_{w}}-{\mu }_{{C}_{y}}{C}_{{y}_{w}}-{\lambda }_{{C}_{y}}{C}_{{y}_{w}},\\ {\dot{T}}_{{h}_{w}} & = & {\lambda }_{{C}_{y}}{\lambda }_{{T}_{h}}{C}_{{y}_{w}}-{\mu }_{{T}_{h}}{T}_{{h}_{w}}-\beta {T}_{{h}_{w}}{S}_{w}+d{T}_{{h}_{f}}-e{T}_{{h}_{w}},\end{array}$$

$$t=\tau \{\begin{array}{c}{S}_{{f}_{\tau }}={S}_{{0}_{f}},{R}_{{f}_{\tau }}=0,\\ {T}_{{r}_{{f}_{\tau }}}=0,{P}_{{r}_{{f}_{\tau }}}=0,{C}_{{y}_{{f}_{\tau }}}=0,{T}_{{h}_{{f}_{\tau }}}={T}_{{h}_{{0}_{f}}},\\ {S}_{{w}_{\tau }}=n({S}_{{w}_{\tau -1}}+{R}_{{w}_{\tau -1}})+{S}_{{w}_{\tau -1}},{R}_{{w}_{\tau }}={R}_{{w}_{\tau -1}},\\ {T}_{{r}_{{w}_{\tau }}}={T}_{{r}_{{w}_{\tau -1}}},{P}_{{r}_{{w}_{\tau }}}={P}_{{r}_{{w}_{\tau -1}}},\\ {C}_{{y}_{{w}_{\tau }}}={C}_{{y}_{{w}_{\tau -1}}},{T}_{{h}_{{w}_{\tau }}}={T}_{{h}_{{w}_{\tau -1}}},\end{array}$$

in which the parameters are the same as in models (1) and (2).

Farming system B considers a flow-through farm with 2 age-class compartments: juvenile fish ${S}_{{f}_{1}}$ and ${R}_{{f}_{1}}$ were maintained in compartment 1 during the first 100 days of the production cycle, these were then transferred into compartment 2 as adult fish ${S}_{{f}_{2}}$ and ${R}_{{f}_{2}}$ during the last 100 days of the production cycle, and eventually harvested from the farm (Fig. 3B). Based on model (3), the farmed fish population was divided into two subpopulations with identical dynamics and interactions with the parasite. It was assumed that there was a continuous water flow from the wild environment to compartment 1, from compartment 1 to compartment 2, and from compartment 2 back to the wild environment. The three transfers of the parasite via this water exchange were represented by the daily rates *e*, *d*_1_ and *d*_2_, respectively.
